# Population genetic differentiation and genomic signatures of adaptation to climate in an abundant lizard

**DOI:** 10.1038/s41437-022-00518-0

**Published:** 2022-03-11

**Authors:** Maravillas Ruiz Miñano, Geoffrey M. While, Weizhao Yang, Christopher P. Burridge, Daniele Salvi, Tobias Uller

**Affiliations:** 1 Discipline of Biological Sciences, University of Tasmania, Hobart, Tas 7005 Australia; 2 Department of Biology, Lund University, Sölvegatan 37, 223 62 Lund, Sweden; 3 Department of Health, Life and Environmental Sciences, University of L’Aquila, Via Vetoio, 67100 Coppito, L’Aquila Italy

**Keywords:** Genetic variation, Genetic variation

## Abstract

Species distributed across climatic gradients will typically experience spatial variation in selection, but gene flow can prevent such selection from causing population genetic differentiation and local adaptation. Here, we studied genomic variation of 415 individuals across 34 populations of the common wall lizard (*Podarcis muralis*) in central Italy. This species is highly abundant throughout this region and populations belong to a single genetic lineage, yet there is extensive phenotypic variation across climatic regimes. We used redundancy analysis to, first, quantify the effect of climate and geography on population genomic variation in this region and, second, to test if climate consistently sorts specific alleles across the landscape. Climate explained 5% of the population genomic variation across the landscape, about half of which was collinear with geography. Linear models and redundancy analyses identified loci that were significantly differentiated across climatic regimes. These loci were distributed across the genome and physically associated with genes putatively involved in thermal tolerance, regulation of temperature-dependent metabolism and reproductive activity, and body colouration. Together, these findings suggest that climate can exercise sufficient selection in lizards to promote genetic differentiation across the landscape in spite of high gene flow.

## Introduction

Understanding how the spatial distribution of environments shapes population differentiation is a shared goal of biogeography, ecology, and evolutionary biology. The balance between selection and gene flow determines the extent to which populations will diverge genetically along environmental gradients and exhibit adaptation to local environmental conditions (Savolainen et al. 2013; Tigano and Friesen 2016; Yeaman and Otto 2011). Local adaptation is common in nature (e.g., Fraser et al. 2011; Halbritter et al. 2018; Hargreaves et al. 2020) and can occur even with high levels of gene flow if selection is strong (Yeaman and Otto 2011; Savolainen et al: 2013; Tigano and Friesen 2016). When phenotypic variation is polygenic, populations can exhibit pronounced adaptive phenotypic divergence with weak geographic genetic differentiation (Yeaman 2015). However, spatial selection mosaics in regions with high gene flow will also tend to promote a genetic architecture with a few divergent genes of large effect (Yeaman and Whitlock 2011), and these genes can therefore exhibit strong genetic differentiation across the landscape.

Climatic gradients commonly impose strong divergent selection across the landscape and can promote adaptive genetic differentiation (Keller et al. 2013; Halbritter et al. 2018). Empirical studies of plants and animals have identified population genetic differentiation across climatic regimes, even over small geographic scales, and loci that reliably associate with temperature, precipitation, and seasonality (e.g., Yeaman et al. 2016; Gibson and Moyle 2020). Adaptive divergence is facilitated when there are severe restrictions on dispersal imposed by geography (e.g., terrestrial vertebrates on islands; Bassitta et al. 2021). However, divergence can also occur in the absence of such barriers if dispersal distances are short, which is commonly the case in small vertebrates like lizards (Clobert et al. 2012; Olsson and Shine 2003; Warner and Shine 2008). As a result, even highly abundant and continuously distributed lizard species can show substantial variation in morphology and colouration across climatic regimes (Ortega et al. 2019; Ruiz Miñano et al. 2021). Furthermore, common garden experiments suggest that local physiological adaptation along climatic gradients is very common (Pettersen 2020). In line with these phenotypic effects, genotype-environment associations have been able to identify genetic differentiation of particular loci associated with climate (e.g., Rodríguez et al. 2017; Prates et al. 2018; Farleigh et al. 2021; see also Campbell-Staton et al. 2016, 2017, 2020). Yet, it remains poorly understood to what extent climate is able to cause genome-wide population differentiation when the opportunity for gene flow is high.

**Fig. 1:**
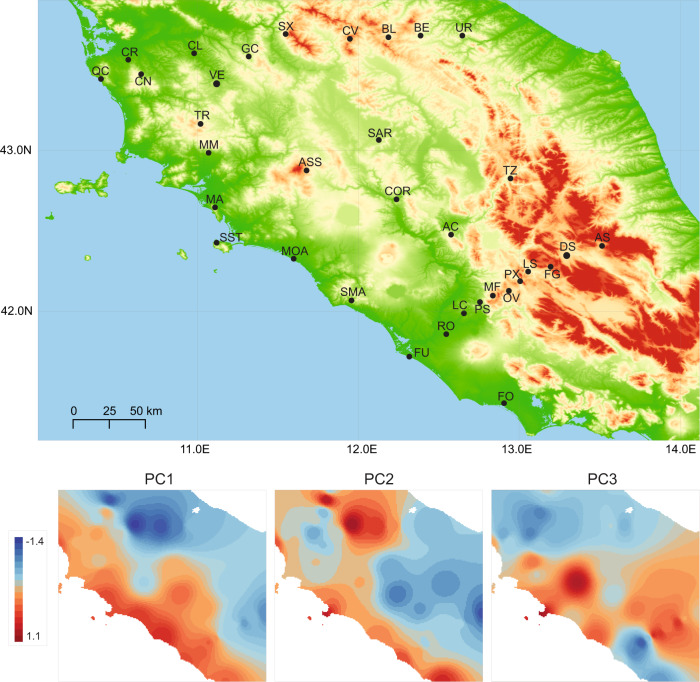
Sampling locations in Italy and their climatic regimes. Top panel: Sampling locations in central Italy (see Table S1 for population acronyms and geographic information). Bottom panels: The spatial distribution of three climatic principal components scores, interpolated from values at the sampling locations.

Common wall lizards (*Podarcis muralis*) in central Italy are well suited to quantify the degree of population genetic differentiation across climatic regimes in the absence of physical barriers to gene flow, and to investigate if local adaptation is accompanied by differentiation at particular loci. The climatic gradients in central Italy can be steep, with a hot, dry Mediterranean climate on the western coast and Tuscan hills, transitioning into a cooler and wetter climate in the Apennine Mountains (Fig. 1; Ruiz Miñano et al. 2021). *Podarcis muralis* is highly abundant throughout this region, but is absent from the hottest and driest locations and occurs only sparsely above 2000 metres elevation (Sindaco et al. 2006). There are several reasons to expect that common wall lizards are experiencing strong local selection as a result of climate. Firstly, climate commonly imposes selection on reproductive biology with lizards typically differing consistently in reproductive characteristics across latitudes and altitudes (e.g., Telemeco et al. 2010; Horvathova et al. 2013; reviewed in Uller and While 2015). Secondly, cool climate imposes selection on the thermal physiology of embryos with, for example, developmental rate evolving in response to modest climatic differences (Oufiero and Angilletta 2006; reviewed in Pettersen 2020) and very rapidly (including in *P. muralis*; Feiner et al. 2018; While et al. 2015b).

In addition to the patterns of adaptive divergence in thermal physiology and life history under natural selection, common wall lizards in this region also show remarkable (genetically determined) variation in body size, shape, and colour. This variation likely results from climatic effects on the strength of sexual selection (or the balance between sexual and natural selection; MacGregor et al. 2017; While et al. 2015a; Yang et al. 2020; Ruiz Miñano et al. 2021). Highly exaggerated colours, large heads, and heavy bodies occur in hot and dry areas, whereas lizards in cooler, more seasonal climate are lighter and exhibit the brown, dull, phenotype characteristic of other genetic lineages of the same species (Ruiz Miñano et al. 2021). Yet, it is currently unknown to what extent these patterns of local phenotypic adaptation to climate are accompanied by genetic differentiation.

To quantify the extent to which climate has caused population genetic differentiation across the landscape, we used reduced representation sequencing and distance-based spatial models. We first investigated the evidence for genetic structure according to climate and geography, and quantified their unique and shared contribution to the overall genetic variation within this region. Second, we used genotype-environment association (GEA) analyses to investigate the evidence for, and distribution of, putative signatures of climatic selection in the genome.

## Methods

### Study system and data collection

The common wall lizard (*Podarcis muralis*) is a small lizard occurring throughout southern Europe (Schulte 2008). While this species has a monotypic brown phenotype across most of its range, there is a pronounced phenotypic variation across central Italy, especially in Latium, Tuscany, and Umbria (Böhme 1986; Ruiz Miñano et al. 2021). This has stimulated the description of a number of subspecies (Böhme 1986), but it is now clear that lizards throughout this area belong to a single evolutionary lineage (the ‘Tuscan’ or ‘[Central] Italian’ lineage, here referred to as the ‘IT’ lineage; Yang et al. 2018, 2022). In the northwest of the IT lineage’s distribution, there is a well-characterised hybrid zone with the distantly related ‘Southern Alps’ lineage and, further north, the river Po forms a natural barrier to gene flow with other lineages (for details, see Yang et al. 2018, 2020, 2022).

Between 2012 and 2018, we collected tail-tip tissue samples of common wall lizards from 71 locations across central and northern Italy (Fig. 1; Table S1; see below and Ruiz Miñano et al. 2021). We refer to each location as a ‘population’. The genomic data for each population was derived from up to fifteen individuals (typically 14; seven females and seven males), using double-digest restriction site-associated DNA sequencing (ddRAD-Seq) on an Illumina HiSeq 2500 platform (Novogene; Hong Kong). Library preparation for ddRAD-Seq data was implemented following the protocol in Peterson et al. (2012) and Yang et al. (2018). In brief, DNA was digested by the restriction enzymes EcoRI High-Fidelity and MspI (New England Biolabs, USA; no methylation sensitive). We used 500 ng of DNA for each individual and amplified with Q5 High-Fidelity DNA Polymerase at selected size of 300–700 bp (New England Biolabs, USA) with 12 cycles. The libraries were distributed and sequenced paired-end across a total of five runs with read length of 150 bp. We obtained approximately 1 Gb for each sample, with expected read depth of 30×. Firstly, sequence reads with low-quality score (Phred score <30), ambiguous base calls, and incomplete barcode calls were removed using “process_radtag” module in STACKS v2.41 (Catchen et al. 2013). We used STACKS to obtain single nucleotide polymorphisms (SNPs) using “ref_map” pipeline (Catchen et al. 2013) by mapping clean reads to the reference genome of *P. muralis* (PodMur_1.0; Andrade et al. 2019). The total assembly of the reference genome is 1.51 Gb with 2162 contig, the N50 size is 92.4 Mb, and the L50 size is 7. SNPs were estimated under a Marukilow model (Maruki and Lynch 2017) with *p*-value 0.05. SNPs with depth <10 were removed as low‐depth loci, and SNPs with depth >95th percentile were removed to avoid PCR duplicates, possible paralogs, and SNPs from high complexity regions (Fan et al. 2016). The individuals with average depth of coverage of <10 for all SNPs were also removed from the data set. PLINK (Chang et al. 2015) was used to filter the SNPs with minimum minor allele frequency (MAF) < 0.05, and with missing rate > 0.1 in each population. Finally, we excluded individuals with genotyping rate < 70% across loci.

Given that our aim was to study population genetic structure within the IT lineage, we first removed all individuals that showed any sign of introgression from other lineages (see Yang et al. 2018; Ruiz Miñano et al. 2022). To this end, we retained only the first SNP per RAD locus (reducing the data from 103,918 SNPs to 35,227 SNPs) and quantified the probability of being assigned to the IT lineage based on ADMIXTURE with *K* = 2. We subsequently removed all individuals whose probability of being assigned to the IT lineage was <0.99. This resulted in the retention of 415 individuals from 34 populations (Fig. 1), all belonging to the IT lineage, with an average of 12 (range 4–15; see Table S1) individuals genotyped per population. These individuals were subsequently subject to spatial analyses with distance-based Moran Eigenvector Maps (dbMEMs) and genotype-environment associations as described in detail below.

### Climatic variables

We ran principal component analyses on 19 bioclimatic variables extracted from WorldClim 2.0 (Fick 2017) at a 30 arc-sec spatial (~1 km^2^) resolution for the 34 sampling locations analysed. The first three axes explained 90% (51.5, 20.4, and 17.7%, respectively) of the variation (Fig. 1 illustrates these with spatial interpolation, using the idw function in the rspatial package; Table S2). PC1 represents a gradient from hot and dry (high values) to cool and wet (low values) climate, and the geographic distribution of the scores clearly separates lowland regions from mountains (Fig. 1). High values for PC2 represent locations with a consistently high temperature across the year (i.e., mild winters and hot summers) and high precipitation seasonality (wet winters and dry summers), which results in a pronounced shift in PC2 from the southwest towards the northeast (Fig. 1). Values for PC3 capture variation that represents locations that are relatively cool and dry (high values) to warm and wet (low values; Fig. 1). These principal components adequately capture the Köppen–Geiger climate classification for Italy (Beck et al. 2018) and were used in all further analyses.

### Spatial genetic structure

We investigated the spatial structure of genetic variation using the data set derived from a single SNP per RAD locus. We used multivariate regression models, redundancy analyses (RDAs), and distance-based Moran Eigenvector Maps (dbMEM). The methods are explained by Legendre and Legendre (2012), and all analyses were carried out in R using the package adespatial (Dray et al. 2021; see Borcard et al. 2018). Redundancy analyses are an extension of linear regression models for multivariate response variables and are suitable for the analyses of genomic data (Forester et al. 2018). dbMEM variables are the eigenvectors of a spatial matrix calculated from the geographical coordinates of the sampling locations (Dray et al. 2006; Legendre and Legendre 2012). These eigenvectors represent orthogonal spatial descriptors of the genetic variation across sampling locations at different spatial scales (defined by Moran’s I). dbMEMs variables can therefore be used as spatial explanatory variables in multiple regression or redundancy analysis of genetic variation.

We created a matrix of geographic distance between locations using the coordinates of each sampling location. Locations were linked within a minimum geographic distance using the longest edge in a minimum spanning tree (Fortin and Dale 2005). We calculated allele frequencies per population and transformed the data using Hellinger transformation (Borcard et al. 2018). This transformation consists of dividing each value in the data matrix by its row sum and taking the square root of the quotient, and thus gives low weight to variables with low counts or lots of zeros (i.e., rare alleles). Isolation by distance (spatial autocorrelation) is expected for population genetic data and this relationship should be removed before further analysis of dbMEMs (Legendre and Legendre 2012). We removed the linear trend (i.e., isolation by distance) by running a linear model of allele frequencies regressed on geographical coordinates and used the residuals for further analyses (Legendre and Legendre 2012). We subsequently ran a global RDA on the detrended population genetic data and used forward selection to identify significant dbMEMs (Blanchet et al. 2008). The significant dbMEMs were subsequently used as independent variables in the final RDA of the genetic data, resulting in one or more canonical axes that represent the spatially structured variation of population allele frequencies across the landscape. We then tested whether these canonical axes were associated with our climatic predictors (PC1, PC2, and PC3) using linear regressions. In addition, we performed a variance partitioning analysis (Legendre and Legendre 2012; Peres-Neto et al. 2006). This method is implemented in the vegan package (Oksanen et al. 2019) and uses partial redundancy analyses to quantify the unique and shared fractions of the genetic variation explained by the three main predictors: the linear (i.e., isolation by distance) and non-linear (i.e., dbMEMs) spatial structure, and climate (i.e., the three climatic PCs). Since the linear trend should be included in the variance partitioning, the data was not detrended before analysis (Legendre and Legendre 2012). Statistical significance and unique and shared contributions of isolation by distance, non-linear spatial structure, and climate were determined by ANOVA using 999 permutations (Borcard et al. 2018).

### Genotype-environment associations

Analysis of genotype-environment association (GEA) aims to detect polygenic responses to selection by investigating changes in allele frequencies (Brauer et al. 2018; Forester et al. 2018). To identify outliers associated with the three climatic principal components, we filtered the 103,918 SNPs obtained after quality control (described above) for the 415 individuals with respect to linkage disequilibrium (LD). To ensure that our results were robust, we ran the subsequent analyses for two conservative cut-offs for LD: r^2^ = 0.8 and r^2^ = 0.5.

We used two different GEA approaches to identify SNPs associated to climate: latent factor models (LFMM; Frichot et al. 2013) and redundancy analyses (Bourret et al. 2014). Both LFMM and RDA assume linear relationships between genetic data and environmental variables (for a methodological review, see Forester et al. 2018). LFMM is a univariate approach that identifies associations between a single locus and environmental variables, using latent factors to correct for confounding effects. We used the *lfmm* package (Caye et al. 2019) to run latent factor models. As population structure is known to be an important confounding factor when detecting genotype associations (Caye et al. 2019), we first investigated the evidence for population structure using principal component analyses, and by estimating the number of ancestry components using a sparse non-negative matrix factorization algorithm (function *snmf* in the *LEA* package; Frichot and Francçois 2015). We tested *K* values ranging from one to ten, plotted the cross-entropy values, and selected *K* based on the inflection point. We calculated genomic inflation factor (GIF) and adjusted the false discovery rate using a Benjamini-Hochberg algorithm (François et al. 2016).

The approach based on RDA detects signatures of selection as a function of a multivariate set of predictors (Forester et al. 2018). Redundancy analyses were performed using the RDA function from the *vegan* package. For this method, we modelled allele frequency as a function of the three climatic PCs and produced three constrained axes (the same as the number of climatic predictors). We identified outlier loci on the constrained axes based on the “locus score”, which represents the loading of each locus in the ordination space. We calculated the mean locus score across all loci for each significant (i.e., *p* < 0.05) RDA axis, and individual loci with a score greater than 3.5 standard deviations from the mean were considered candidates for selection (Forester et al. 2016). Since the RDA approach is more powerful when not including factors that account for population structure (Forester et al. 2018), we did not include any such variable in the final model. We assessed multicollinearity between variables (climatic variables and dbMEMs) using the variation inflation factor (VIF); all VIF were <2 and therefore all were retained in the final model. All the statistical analyses were performed in R v. 4.0.2.

To infer the potential function for candidate genes, we conducted enrichment analyses based on Gene Ontology (GO) annotation using clusterProfiler package with default parameters (Yu et al. 2012) in R, for the outliers identified by RDA (which is the method considered to deliver the most robust list of candidates; Forester et al. 2018) and for the final outlier data set representing SNPs found by both LFMM and RDA approaches.

## Results

### Genetic differentiation across climatic regimes

After stringent filtering for sequencing depth and rate of missing data, and excluding all but the first SNP per RAD locus, 35,227 SNPs were retained. Expected heterozygosity, observed heterozygosity, and inbreeding coefficients were similar across populations (e.g., average expected heterozygosity, H_E_ = 0.199, range 0.181–0.209; see Table S3), and uncorrelated with any of three climatic PCs (all *p* > 0.05). The global F_st_ for the 34 populations was 0.0615 (CI: 0.0606 – 0.0642 from 100 iterations of bootstrap).

**Fig. 2:**
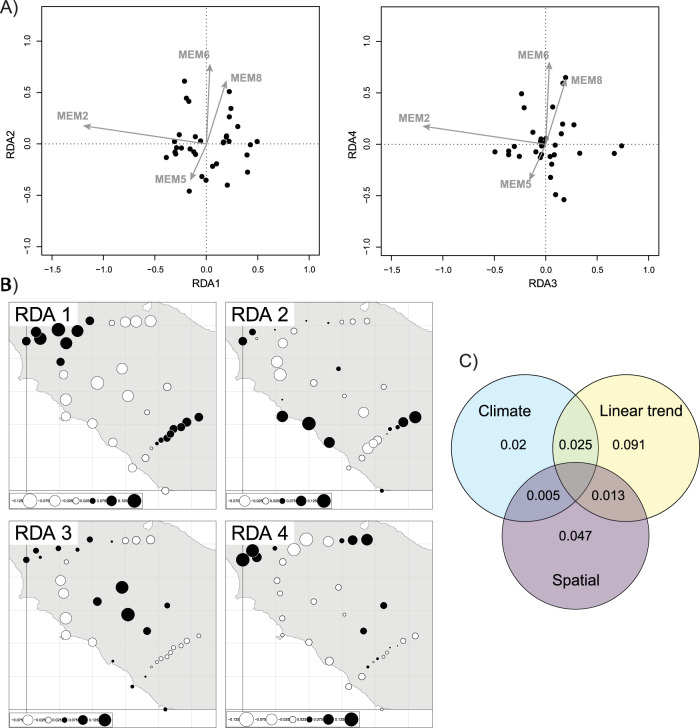
Results from the dbMEM analyses and variance partitioning of the allele frequency data. **A** RDA triplots of the Hellinger transformed allele frequency data. Dots represent sampling locations and arrows the four dbMEMs retained following forward selection. **B** dbMEM fitted scores for the four significant canonical axes of the redundancy analysis. Black dots represent positive values and white dots negative values. **C** Variance partitioning of the allele frequency data with unique and shared fractions of explained variation.

The Hellinger transformed population allele frequencies showed a significant linear association with geographic coordinates (F_2,31_ = 2.92, *P* < 0.001; Fig. S1). The data were therefore detrended for the subsequent analysis of spatial structure using dbMEMs (Legendre and Legendre 2012). After forward selection, four dbMEMs describing population allele frequencies were retained (*P* ≤ 0.05; Table S4; Fig. S2, S3). A redundancy analysis on these four significant dbMEMs as response variables resulted in four canonical axes that together explained 6.0% of the variation (F_4,29_ = 1.52, *P* < 0.001; Fig. 2; Table 1). The first two canonical axes describe a rather broad-scale spatial structure in allele frequencies and the other two finer scale spatial structure (Fig. 2). None of these canonical axes were significantly associated with the climatic predictors (linear regressions with PC1, PC2, and PC3 as predictors; Table S5).

**Table 1:** Redundancy analysis of the detrended genetic data with the four significant dbMEMs.

Coefficients	df	Variance	F	*P*-value
RDA1	1	0.009	2.15	0.001
RDA2	1	0.006	1.39	0.027
RDA3	1	0.005	1.32	0.034
RDA4	1	0.005	1.22	0.045
Residual	29	0.116		

Significance determined using ANOVA with 999 permutations; Full model: F_4,29_ = 1.52, *P* = 0.001; Adjusted R^2^ = 0.059.

Variance partitioning analyses makes it possible to assess the unique and shared fraction of genomic variation explained by the three main predictors: the linear trend, spatial structure (i.e., four dbMEMs), and climate variables (i.e., three climatic PCs). The total amount of variation in the Hellinger transformed population allele frequencies explained by the linear trend, dbMEMs and climate was 17.6%. An RDA on the non-detrended (i.e., without removing linear trend) Hellinger transformed allele frequencies, using forward selection of climatic principal components, resulted in retention of climatic PC1 and PC2. These two predictors uniquely explained about 2% of the variation in allele frequencies (R^2^_adj_ = 0.02, F _2,25_ = 1.33, *P* = 0.002; Fig. 2). An additional 3% of variation explained by climate was collinear with the linear trend or dbMEMs. The spatial structure (linear trend and dbMEMs) alone accounted for a total of 15.1 % of variation in the Hellinger transformed genetic variation, with the unique proportions of variance being statistically significant for both the linear trend (R^2^_adj_ = 0.091, F _2,25_ = 2.49 *P* < 0.001; Fig. 2) and the non-linear spatial structure captured by the dbMEMs (R^2^_adj_ = 0.047, F _4,25_ = 1.41, *P* < 0.001; Fig. 2).

### Genotype-environment associations

After filtering on LD at r^2^ = 0.8, we retained 80,285 SNPs for the analyses of genotype-environment associations. Using the more stringent filtering of r^2^ = 0.5 resulted in ~10% fewer SNPs (72,350), but a very high overlap in terms of significant outlier SNPs following LFMM and RDA (see below). This suggests that the results are robust at varying cut-offs, and we therefore report here only the results for the data set filtered at r^2^ = 0.8.

A principal component analysis and the broken stick method did not support a significant genetic clustering (Fig. S4), but sNMF identified six clusters (Fig. S5). We therefore set *K* = 6 in the LFMM analyses. The LFMM identified 221 candidate loci, of which 75 were associated with climatic PC1, 57 with PC2, and 89 with PC3. Manual adjustment of the genomic inflation factors (GIF) had only minor effect on these numbers.

The RDA returned three significant axes, on which we identified 820 unique candidate loci. The majority of these SNPs were most strongly associated with climatic PC1 (361), followed by PC3 (247) and PC2 (212). GO enrichment for these RDA outliers can be found in Table S6.

Out of these candidate SNPs, 48 were detected by both LFMM and RDA (the corresponding number for LD filtering at r^2^ = 0.5 was 43, of which 38 were shared with results for r^2^ = 0.8). These 48 outliers were dispersed throughout the genome, found on 16 of the 19 chromosomes; 23 outliers were most strongly associated with climatic PC1, 11 with PC2, and 14 with PC3. These SNPs were associated with 37 annotated genes (see Discussion and Table S7). The GO enrichment for these genes identified molecular functions related to catabolic processes (GO:0044248, GO:1901575, and GO:0009056), metabolic processes (GO:0044237 and GO:0008152), and oxidoreductase activity (GO:0016491).

## Discussion

Quantifying the extent of climate-associated population genetic differentiation, and its spatial scale, is important for understanding past and future adaptation to climatic regimes. In this study, we investigated the relationship of genome-wide SNPs with climatic and spatial data across 34 populations of the common wall lizard (*Podarcis muralis*) in central Italy. This region is characterised by very high lizard densities, no physical barriers to gene flow, consistently high estimates of population genetic diversity (even at climatic extremes), and a genetic structure characterized by isolation by distance (Ruiz Miñano et al. 2021; Yang et al. 2018). Overall, our results demonstrate that climatic selection is sufficient to overcome high levels of gene flow and cause genetic differentiation, consistently sorting particular alleles across the landscape according to climatic regimes. As a result, we expect wall lizards to show pronounced local adaptation in morphology, physiology, and behaviour even across relatively small spatial scales.

In support of previous work (Yang et al. 2018), most of the genetic structure within central Italy, accounting for 12.9% of the total population genomic variation, represents a linear trend that is consistent with isolation by distance. The fine- to medium-scale spatial structure was less pronounced (uniquely explaining ~4.5% of population genetic variation), which is consistent with the lack of physical barriers to gene flow and absence of historical isolation of allopatric populations within the region (Yang et al. 2022). Excluding the additional variation explained by climate (see below), it is therefore perhaps not surprising that >80% of population genomic variation remained unexplained. Nevertheless, we cannot exclude that some of this unexplained variation is caused by historical or more recent gene flow from peripheral populations or lineages (Yang et al. 2022).

Our emphasis in this study was to test if the climatic variation within this region contributed to genome-wide patterns of genetic diversity and population differentiation. While there was no evidence that populations at climatic extremes have lower genetic variation, climate explained about 5% of the population genomic variation across the 34 populations. This result contributes to a growing literature on climate-associated genome-wide population differentiation in lizards (e.g., Rodríguez et al. 2017; Farleigh et al. 2021; for a study testing non-climate environmental influence on genome wide population differentiation of three species of lizard, see Krohn et al. 2019). While these studies do support a role for climate in shaping genetic differentiation, they are also somewhat limited by virtue of being largely descriptive. Further work would therefore benefit from sampling at spatial scales and across climatic regimes that allow more explicit tests of, for example, the diluting effect of gene flow on adaptive population divergence. Lineages or species that evolve along similar climatic gradients would also be interesting contrasts, in particular for identifying the extent to which climatic adaptation makes use of similar genes and pathways (e.g., Feiner et al. 2018).

About half of the genomic variation explained by climate was collinear with a linear shift in allele frequencies. This collinearity reflects the transition from a temperate, hot, and dry climate of the south-west coast, to a more oceanic climate with no dry season in the Apennine mountains and in the north-east of the distribution of the IT lineage of *P. muralis* (see Yang et al. 2022 for the phylogeography of this species). The two percent of genomic variation explained uniquely by climate represents fine- to medium-scale climatic variation not captured by either the linear or non-linear spatial structure. Common wall lizards are extremely abundant throughout central Italy and inhabit a wide range of microhabitats except for the hottest and driest environments (where the congener *P. siculus* entirely takes over; Capula et al. 1993). Future studies should test the extent to which the unexplained genetic variation could be accounted for by additional landscape features (e.g., habitat type, presence of competitors, human habitation; Beninde et al. 2016; Van Buskirk and Jansen van Rensburg 2020).

Common garden experiments and reciprocal transplants of lizards between climatic regimes have established that growth and other fitness proxies are commonly highest in the local environment (e.g., Niewiarowski and Roosenburg 1993; Uller and Olsson 2003; Iraeta et al 2006; While et al. 2015b). For example, reproductive biology and developmental rates can be locally adapted to the length of the activity season and the thermal conditions during spring and summer (reviewed in Uller and While 2015; Pettersen 2020). These traits provide a wide range of targets for selection that can contribute to population genetic differentiation. The ontology of genes associated with outlier SNPs is rather uninformative since the categories (e.g., metabolic activity) are broad and difficult to link to a priori expectations. However, a few of the most robust outlier SNPs (i.e., those identified by multiple methods) were associated with candidate genes that functional studies have shown to be important to thermal physiology. These include CDK4, a cyclin-dependent kinase, that is repressed in response to freezing temperatures in frogs (Zhang and Storey 2012), and a member of the heat shock protein family (HSPA8), which is a constitutively expressed chaperone that has been shown to have a seasonal expression pattern in heat- and cold-adapted goat breeds (Banerjee et al. 2014; Singh et al. 2017).

We may also expect to identify genes involved in regulation of development, metabolism, growth, and biological cycles, such as annual reproductive activity. The dual oxidase 2 (DUOX2) and its accessory protein (DUOXA2), both located on chromosome 14, are putative candidates since they are involved in thyroid hormone biosynthesis (Carvalho and Dupuy 2013). Thyroid hormone has been shown to mediate the effects of temperature on reptile reproductive physiology (Norris and Lopez 2011) and regulate embryonic growth and development (Ruuskanen and Hsu 2018). For example, experimental manipulation of thyroid hormones *in ovo* can modify the time to hatching in turtles and birds (e.g., McGlashan et al. 2017; reviewed in Ruuskanen and Hsu 2018). Thyroid hormone regulation is therefore likely to play important roles in climate adaptation in ectotherm vertebrates, including high developmental rate in cool-adapted populations (Pettersen 2020); an adaptation demonstrated to evolve rapidly in *P. muralis* (While et al. 2015b; Feiner et al. 2018).

It is not surprising to find candidate genes that are consistent with the expectation that climatic selection will lead to population divergence in genes that regulate thermal physiology and annual life cycles. In common wall lizards, climate is also associated with a marked shift in several morphological characters that are under sexual selection, including head size, shape, and colour ornamentation (While et al. 2015a; Ruiz Miñano et al. 2021). Theoretical studies suggest that exaggerated armaments and ornaments could be explained by an increased opportunity for female monopolization by dominant males in climates that support a long reproductive season and less synchronous female reproduction (reviewed in Shuster and Wade 2003; García-Roa et al. 2020). This adds another selective pressure that varies across the landscape in association with climate, and could contribute to divergence of genes that may at first appear of little relevance for climate adaptation per se. One of the candidate genes identified here, RAB7A, may reflect this climatic effect on sexual selection, and in particular sexual ornamentation. This gene encodes a Rab GTPase that regulates transport of early and intermediate melanosomes (Gomez et al. 2001; Fukuda 2021), the melanin-containing organelles of the melanophores in lizard skin (Taylor and Hadley 1970). RAB7A may also be involved in guanine synthesis or transport in iridophores (Stuckert et al. 2019), another cell type that can be responsible for the difference between brown and green skin in lizards (Eisentraut 1950; Kuriyama et al. 2017; Kuriyama et al. 2020). Thus, we hypothesize that this gene is involved in the expression of the conspicuous black and green colour ornamentation (i.e., “*P. muralis nigriventris”*; Böhme 1986) that is closely associated with coastal climate in *P. muralis* from western central Italy (Ruiz Miñano et al. 2021).

It is encouraging to find candidate genes that have previously been shown to be involved in both thermal physiology and animal colouration. However, genotype-environment associations are limited in the extent to which they can inform us about causality. Thus, these candidates remain putative loci of adaptation until quantitative experiments confirm the results. Furthermore, RAD data only covers a small part of the genome and most major effect candidates will therefore go undetected. Nevertheless, the results of this study indicates that climatic selection may affect a broad range of genes dispersed throughout the genome. Whole-genome sequences across steep climatic gradients, accompanied by transitions in phenotype (Ruiz Miñano et al. 2021), could help to further identify such genomic targets of climatic selection.

In summary, this study demonstrates that climate exercises an effect on population genetic differentiation in an abundant lizard with extensive gene flow. Further, the analyses suggest that particular alleles are consistently favoured in certain climatic regimes. More targeted approaches, combining functional studies with whole-genome sequencing, will be necessary to identify additional genes underlying population differentiation and local adaptation.

## Supplementary information

Supplementary Tables and Figures

## Data Availability

All sequence data are available at GenBank, accession number: PRJNA486080. Population level data included as supplementary information (Table S1).
