# Supplementary material for: Population genetic differentiation and genomic signatures of adaptation to climate in an abundant lizard
Source: Heredity (Edinb). 2022 Mar 11;128(4):271–8. doi: 10.1038/s41437-022-00518-0 (PMC8987050; doi:10.1038/s41437-022-00518-0)
Supplement: Supplementary file 1 — Supplementary Tables and Figures [file 41437_2022_518_MOESM1_ESM.docx]

**Supplementary Information**

Population genetic differentiation and genomic signatures of adaptation to climate in an abundant lizard

Maravillas Ruiz Miñano^1,2^, Geoffrey M While^1^, Weizhao Yang^2^, Christopher Burridge^1^, Daniele Salvi^3^ & Tobias Uller^2^

1. Discipline of Biological Sciences, University of Tasmania, Hobart, Tasmania 7005, Australia
2. Department of Biology, Lund University, Sölvegatan 37, 223 62 Lund, Sweden
3. Department of Health, Life and Environmental Sciences, University of L’Aquila, Via Vetoio, 67100 Coppito, L’Aquila, Italy

Supplementary Tables S1-S7

Supplementary Figures

Table S1. Summary of sampling locations and number of individuals genotyped per population. For a map with locations, see Figure S1.

| Population ID | Sampling Location | Latitude | Longitude | # Samples genotyped |
| --- | --- | --- | --- | --- |
| AC | Altrocanto | 42,4807 | 12,736 | 11 |
| AS | Assergi | 42,4144 | 13,5090 | 6 |
| ASS | Abbadia San Salvatore | 42,8813 | 11,6739 | 14 |
| BE | Belforte all'isauro | 43,7168 | 12,3761 | 14 |
| BL | Badia Tedalda | 43,7100 | 12,1813 | 14 |
| CL | Castelfiorentino | 43,6058 | 10,9697 | 11 |
| CN | Chianni | 43,4842 | 10,6425 | 12 |
| COR | Corbara | 42,7033 | 12,2276 | 14 |
| CR | Crespina | 43,5748 | 10,5649 | 14 |
| CV | Chiusi della Verna | 43,6981 | 11,9360 | 14 |
| DS | Colle di Sassa | 42,3468 | 13,2917 | 12 |
| FG | Fagge | 42,2786 | 13,1889 | 7 |
| FO | Fogliano | 41,4133 | 12,9043 | 14 |
| FU | Castel Fusano | 41,7234 | 12,3061 | 13 |
| GC | Greve in Chianti | 43,5876 | 11,3148 | 14 |
| LC | Santa Lucia | 41,9908 | 12,6516 | 10 |
| LS | Lago del Salto | 42,2540 | 13,0521 | 14 |
| MA | Maremma | 42,6535 | 11,1032 | 14 |
| MF | Monteflavio | 42,1030 | 12,8265 | 14 |
| MM | Montemassi | 42,9922 | 11,0639 | 15 |
| MOA | Montalto | 42,3279 | 11,5855 | 14 |
| OV | Orvinio | 42,1300 | 12,9339 | 14 |
| PS | Palombara Sabina | 42,0565 | 12,7512 | 13 |
| PX | Paganico | 42,1867 | 12,9971 | 14 |
| QC | Quercianella | 43,4494 | 10,3892 | 5 |
| RO | Rome (Torre Fiscale) | 41,8586 | 12,5442 | 14 |
| SAR | Sant'Arcangelo | 43,0743 | 12,1206 | 4 |
| SMA | Santa Marinella | 42,0703 | 11,9543 | 6 |
| SST | Santo Stefano | 42,4250 | 11,1050 | 14 |
| SX | Saltino | 43,7267 | 11,5382 | 14 |
| TR | Travale | 43,1674 | 11,0084 | 14 |
| TZ | Triponzo | 42,8278 | 12,9382 | 12 |
| UR | Urbino | 43,7236 | 12,6359 | 14 |
| VE | Colle di Val d'Elsa | 43,4215 | 11,1118 | 13 |

Table S2. Loadings for the first two principal components on the bioclimatic variables. The first three PCs, explaining 89.7% of the variation, were statistically significant in a broken stick model and used in all subsequent modelling.

| **Bioclimatic variable** | **PC1** | **PC2** | **PC3** |
| --- | --- | --- | --- |
| BIO1 = Annual Mean Temperature | **0.29** | 0.10 | -0.13 |
| BIO2 = Mean Diurnal Range (Mean of monthly (max temp - min temp)) | 0.05 | **-0.20** | **-0.48** |
| BIO3 = Isothermality (BIO2/BIO7) (×100) | 0.14 | -0.09 | **-0.44** |
| BIO4 = Temperature Seasonality (SD ×100) | -0.18 | **-0.30** | **-0.24** |
| BIO5 = Max Temperature of Warmest Month | **0.27** | 0.02 | **-0.27** |
| BIO6 = Min Temperature of Coldest Month | **0.28** | **0.22** | 0.08 |
| BIO7 = Temperature Annual Range (BIO5-BIO6) | -0.06 | **-0.28** | **-0.44** |
| BIO8 = Mean Temperature of Wettest Quarter | 0.19 | **-0.23** | 0.02 |
| BIO9 = Mean Temperature of Driest Quarter | 0.18 | **0.23** | -0.18 |
| BIO10 = Mean Temperature of Warmest Quarter | **0.28** | 0.05 | -0.19 |
| BIO11 = Mean Temperature of Coldest Quarter | **0.30** | 0.16 | -0.05 |
| BIO12 = Annual Precipitation | **-0.24** | **0.27** | -0.18 |
| BIO13 = Precipitation of Wettest Month | **-0.20** | **0.34** | -0.19 |
| BIO14 = Precipitation of Driest Month | **-0.30** | -0.01 | -0.05 |
| BIO15 = Precipitation Seasonality (CV) | **0.22** | **0.31** | -0.04 |
| BIO16 = Precipitation of Wettest Quarter | **-0.20** | **0.34** | -0.19 |
| BIO17 = Precipitation of Driest Quarter | **-0.29** | 0.07 | -0.12 |
| BIO18 = Precipitation of Warmest Quarter | **-0.29** | -0.01 | -0.06 |
| BIO19 = Precipitation of Coldest Quarter | -0.13 | **0.42** | -0.18 |

Table S3. Expected heterozygosity (H_E_), observed heterozygosity (H_O_) and inbreeding coefficient (F_IS_) for the populations included in this study, calculated on the basis of 35,227 SNPs. Average H_E_ = 0.199; Average H_O_ = 0.179; Average F_IS_ = 0.060

| Population | H_E_ | H_O_ | F_IS_ |
| --- | --- | --- | --- |
| AC | 0.202 | 0.180 | 0.089 |
| AS | 0.192 | 0.171 | 0.074 |
| ASS | 0.206 | 0.185 | 0.085 |
| BE | 0.195 | 0.177 | 0.076 |
| BL | 0.201 | 0.182 | 0.076 |
| CL | 0.207 | 0.181 | 0.099 |
| CN | 0.205 | 0.184 | 0.081 |
| COR | 0.206 | 0.184 | 0.087 |
| CR | 0.202 | 0.183 | 0.076 |
| CV | 0.207 | 0.188 | 0.077 |
| DS | 0.197 | 0.174 | 0.095 |
| FG | 0.204 | 0.180 | 0.087 |
| FO | 0.185 | 0.162 | 0.105 |
| FU | 0.191 | 0.170 | 0.092 |
| GC | 0.207 | 0.186 | 0.081 |
| LC | 0.203 | 0.183 | 0.075 |
| LS | 0.205 | 0.185 | 0.081 |
| MA | 0.198 | 0.178 | 0.084 |
| MF | 0.203 | 0.181 | 0.089 |
| MM | 0.202 | 0.184 | 0.073 |
| MOA | 0.203 | 0.178 | 0.100 |
| OV | 0.201 | 0.183 | 0.074 |
| PS | 0.204 | 0.183 | 0.086 |
| PX | 0.203 | 0.182 | 0.083 |
| QC | 0.196 | 0.185 | 0.022 |
| RO | 0.197 | 0.177 | 0.084 |
| SAR | 0.199 | 0.180 | 0.048 |
| SMA | 0.198 | 0.177 | 0.072 |
| SST | 0.186 | 0.167 | 0.084 |
| SX | 0.183 | 0.176 | 0.032 |
| TR | 0.209 | 0.190 | 0.074 |
| TZ | 0.191 | 0.175 | 0.067 |
| UR | 0.181 | 0.164 | 0.078 |
| VE | 0.199 | 0.181 | 0.070 |

Table S4. List of dbMEM variables and their contribution to SNP variation as determined by forward variable selection with RDA

| Variables | AdjR^2^ | F | pvalue |
| --- | --- | --- | --- |
| MEM2 | 0.031 | 2.06 | 0.001 |
| MEM5 | 0.01 | 1.3 | 0.015 |
| MEM8 | 0.01 | 1.31 | 0.006 |
| MEM9 | 0.01 | 1.31 | 0.011 |

Table S5. Linear regression of the fitted scores for the four significant axes (RDA1, RDA2, RDA3 and RDA4) on the three climatic principal components in the Italian lineage. RDA1 Full model: F_3,30_ = 1.17, P = 0.34; Adjusted R^2^ = 0.01; Residual error: 0.063; RDA2 Full model: F_3,30_ = 0.66, P = 0.58; Adjusted R^2^ = -0.03; Residual error: 0.052. RDA3 Full model: F_3,30_ = 0.74, P = 0.54; Adjusted R^2^ = -0.024; Residual error: 0.05; RDA4 Full model: F_3,30_ = 1.06, P = 0.38; Adjusted R^2^ = 0.005; Residual error: 0.05.

| Canonical axis | Coefficients | Estimate ± SE | t-value | P-value |
| --- | --- | --- | --- | --- |
| RDA1 | Intercept | 1.6×10^-6^ ± 0.011 | 0 | 1.0 |
|  | PC1 | -0.022 ± 0.017 | -1.25 | 0.22 |
|  | PC2 | -0.012 ± 0.028 | -0.45 | 0.66 |
|  | PC3 | -0.039 ± 0.030 | -1.32 | 0.20 |
| RDA2 | Intercept | -1.6×10^-6^ ± 0.009 | 0 | 1.0 |
|  | PC1 | 0.005 ± 0.014 | 0.37 | 0.71 |
|  | PC2 | 0.009 ± 0.029 | 0.41 | 0.68 |
|  | PC3 | -0.032 ± 0.024 | 1.29 | 0.21 |
| RDA3 | Intercept | 2.54 ×10^-6^ ± 0.009 | 0 | 1.0 |
|  | PC1 | -2.7 ×10^-4^ ± 0.014 | -0.02 | 0.99 |
|  | PC2 | -0.019 ± 0.021 | -0.88 | 0.39 |
|  | PC3 | -0.029 ± 0.024 | -1.20 | 0.24 |
| RDA4 | Intercept | 3.35 ×10^-6^ ± 0.008 | 0 | 1.0 |
|  | PC1 | 0.012 ± 0.013 | 0.94 | 0.36 |
|  | PC2 | -0.03 ± 0.021 | -1.44 | 0.16 |
|  | PC3 | -0.011 ± 0.025 | -0.48 | 0.63 |

Table S6. GO enrichment for RDA outliers.

| ID | Description | pvalue | geneID |
| --- | --- | --- | --- |
| GO:0006508 | proteolysis | 0.00770392 | POMURG00000021269/POMURG00000011092/POMURG00000018875/POMURG00000021897 |
| GO:0050790 | regulation of catalytic activity | 0.00770392 | POMURG00000017484/POMURG00000018875/POMURG00000021791/POMURG00000015494 |
| GO:0065009 | regulation of molecular function | 0.015953141 | POMURG00000017484/POMURG00000018875/POMURG00000021791/POMURG00000015494 |
| GO:0040007 | growth | 0.031920721 | POMURG00000000489/POMURG00000022645 |
| GO:0072593 | reactive oxygen species metabolic process | 0.031920721 | POMURG00000011123/POMURG00000022126 |
| GO:0012505 | endomembrane system | 0.045120392 | POMURG00000017162/POMURG00000022000/POMURG00000022575/POMURG00000023533 |
| GO:0006631 | fatty acid metabolic process | 0.046030463 | POMURG00000014776/POMURG00000023467 |
| GO:0019725 | cellular homeostasis | 0.046030463 | POMURG00000022587/POMURG00000023129 |
| GO:0030246 | carbohydrate binding | 0.046030463 | POMURG00000022587/POMURG00000018706 |
| GO:0048878 | chemical homeostasis | 0.046030463 | POMURG00000022587/POMURG00000023129 |
| GO:0051336 | regulation of hydrolase activity | 0.046030463 | POMURG00000018875/POMURG00000015494 |

Table S7. Candidate genes identified by both RDA and LFMM analyses of genotype-climate associations.

| Gene ID | Gene | Definition |
| --- | --- | --- |
| POMURG00000002230 | MRPL15 | mitochondrial ribosomal protein L15 |
| POMURG00000002231 | TCEA1 | transcription elongation factor A1 |
| POMURG00000002232 | RGS20 | regulator of G protein signaling 20 |
| POMURG00000003090 | GABRG1 | gamma-aminobutyric acid type a receptor subunit gamma1 |
| POMURG00000003091 |  |  |
| POMURG00000003347 | ZNF827 | zinc finger protein 827 |
| POMURG00000003348 | MMAA | metabolism of cobalamin associated A |
| POMURG00000005626 | SF3B1 | splicing factor 3B subunit 1 |
| POMURG00000005627 | ANKRD44 | ankyrin repeat domain 44 |
| POMURG00000006358 |  |  |
| POMURG00000006482 | SORT1 | sortilin 1 |
| POMURG00000006483 | GALR3 | galanin receptor 3 |
| POMURG00000006484 | CPNE8 | copine 8 |
| POMURG00000007009 |  |  |
| POMURG00000007415 |  |  |
| POMURG00000007978 | OLIG1 | oligodendrocyte transcription factor 1 |
| POMURG00000008707 | CTDSP2 | ctd small phosphatase 2 |
| POMURG00000008715 | CDK4 | cyclin-dependent kinase 4 |
| POMURG00000008716 | TSPAN31 | tetraspaini 31 |
| POMURG00000008717 |  |  |
| POMURG00000008718 | AGAP2 | arfGAP with GTPase domain, ankyrin repeat and PH domain 2 |
| POMURG00000009570 | PKD2L2 | polycystin 2 like 2 |
| POMURG00000009571 | FAM13B | family with sequence similarity 13 member B |
| POMURG00000011123 | DUOX2 | dual oxidase 2 |
| POMURG00000011124 | DUOXA2 | dual oxidase a2 |
| POMURG00000011125 |  |  |
| POMURG00000013347 | KCNK12 | potassium two pore domain channel subfamily K member 12 |
| POMURG00000013380 | PNPT1 | polyribonucleotide nucleotidyltransferase 1 |
| POMURG00000013381 | EFEMP1 | EGF containing fibulin extracellular matrix protein 1 |
| POMURG00000014006 |  |  |
| POMURG00000015190 | TF2-6 | transposon Tf2-6 polyprotein |
| POMURG00000015191 | KLF9 | kruppel like factor 9 |
| POMURG00000015192 |  |  |
| POMURG00000015193 |  |  |
| POMURG00000015194 |  |  |
| POMURG00000015195 |  |  |
| POMURG00000015781 |  |  |
| POMURG00000015926 |  |  |
| POMURG00000015927 |  |  |
| POMURG00000017817 | KAT6A | lysine acetyltransferase 6a |
| POMURG00000019240 | TBC1D30 | TBC domain family member 30 |
| POMURG00000021535 |  |  |
| POMURG00000021536 | FEM1A | fem-1 homolog a |
| POMURG00000021537 | TICAM1 | toll like receptor adaptor molecule 1 |
| POMURG00000022007 |  |  |
| POMURG00000022364 | CLMP | CXADR like membrane protein |
| POMURG00000022365 |  |  |
| POMURG00000022366 | HSPA8 | heat shock protein family a member 8 |
| POMURG00000022367 |  |  |
| POMURG00000023446 | PRSS55 | serine protease 55 |
| POMURG00000023447 | SEMA3F | semaphorin 3F |
| POMURG00000023448 | KDM5C | lysine demethylase 5c |
| POMURG00000023489 | RAB7A | member RAS oncogene family |
| POMURG00000023490 | HCFC1 | host cell factor c1 |
| POMURG00000023491 | TMEM187 | transmembrane protein 187 |

Fig. S1. Mantel correlogram for the Hellinger transformed genetic data. The distance on the x-axis is in kilometres. Significant multivariate spatial autocorrelation is indicated by the black squares.

Fig. S2. Scalogram showing the explained variance (unadjusted R^2^) of the Hellinger transformed genetic data explained by the dbMEM eigenfunctions. Colours indicate significance tests based on permutations.

Fig. S3. The four significant dbMEM variables (retained following forward simulation) with positive spatial autocorrelation on the Hellinger transformed genetic data. The MEMs are ordered from broad to fine spatial scale. Plotted are the scores for each population, indicated by the colour and size of the dots (white = negative, black = positive). The grid indicates the geographic distance (d = 50km).


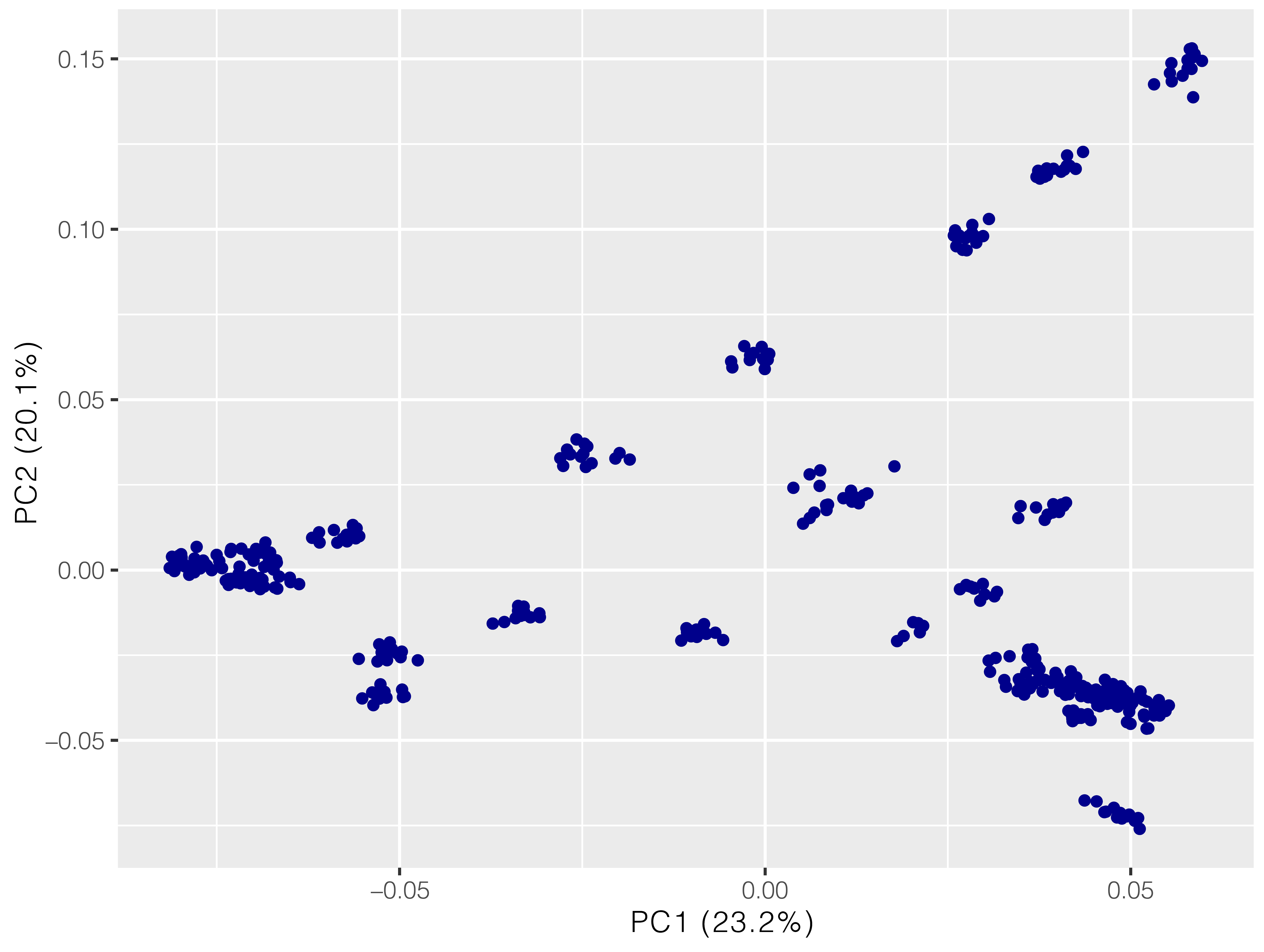


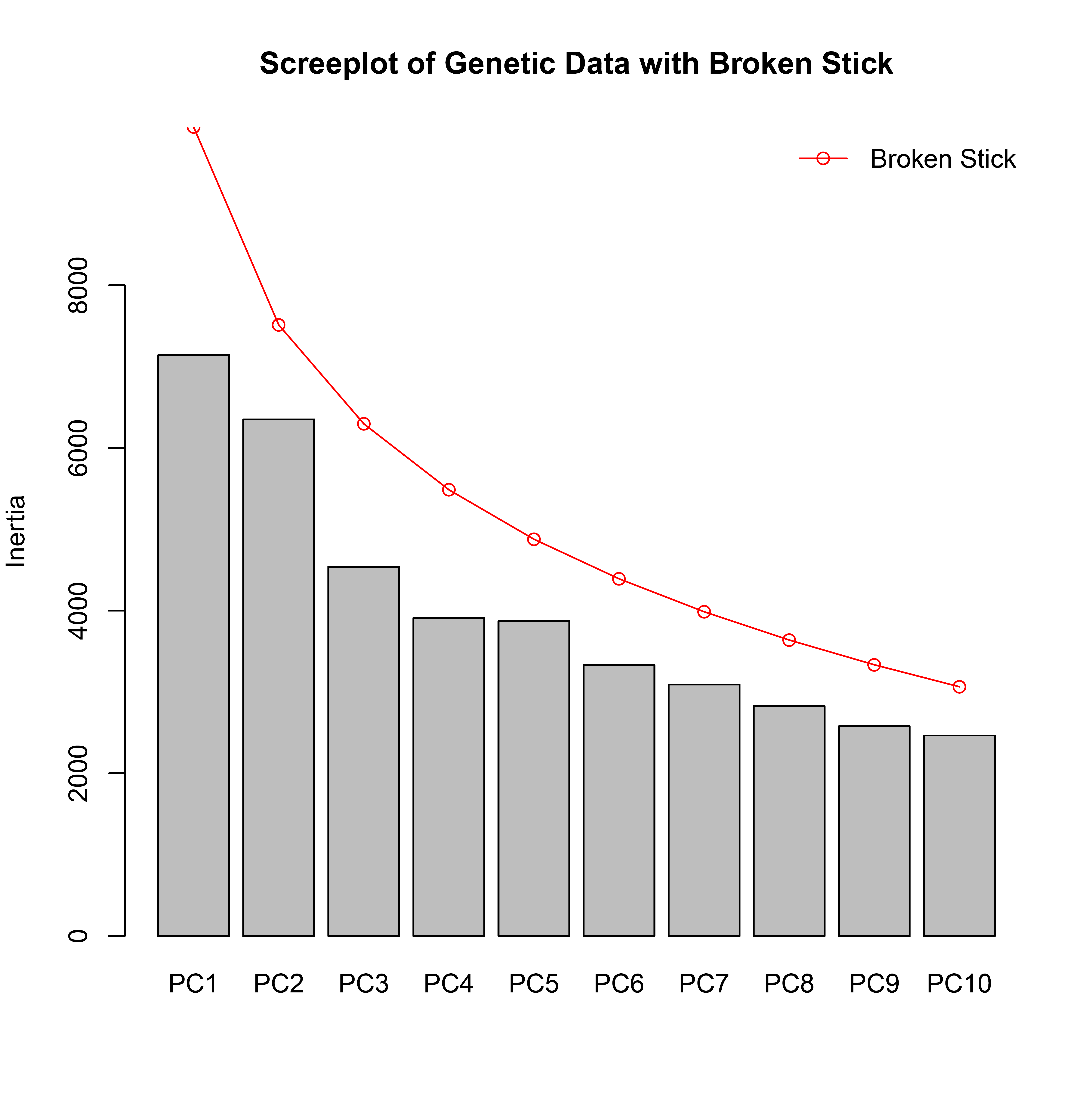


Fig. S4. Principal component analysis on the second genetic data set. Top panel shows the first two principal components (PCs). Bottom panel shows the screeplot using the broken stick method.

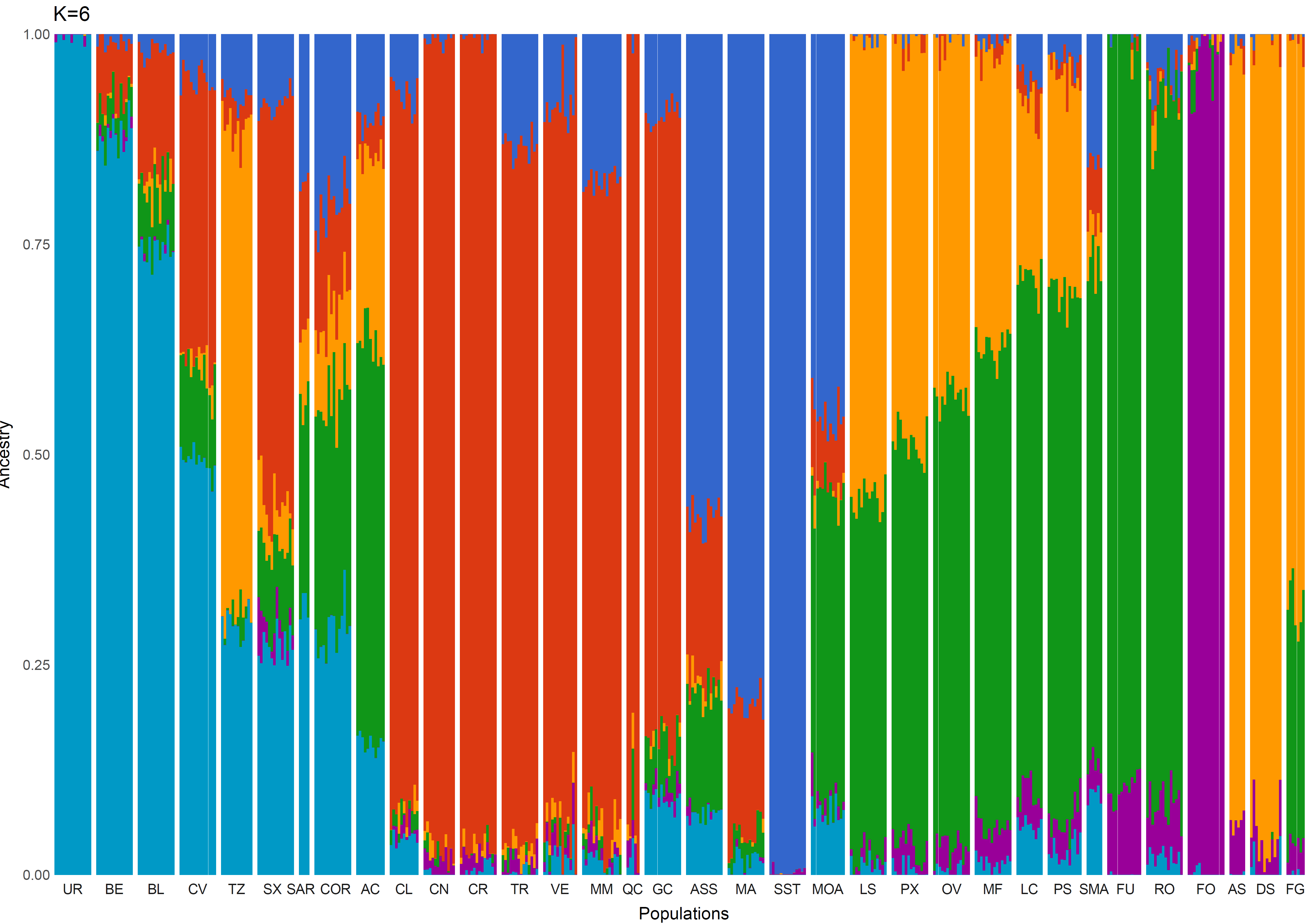


Fig. S5. Results from the sNMF analysis. The top panel shows the values of the cross-entropy criterion and cross-validation. The bottom panel shows the ancestry estimates for each individual, grouped into populations.
